# Broad Phylogenomic Sampling and the Sister Lineage of Land Plants

**DOI:** 10.1371/journal.pone.0029696

**Published:** 2012-01-13

**Authors:** Ruth E. Timme, Tsvetan R. Bachvaroff, Charles F. Delwiche

**Affiliations:** 1 Cell Biology and Molecular Genetics, University of Maryland, College Park, Maryland, United States of America; 2 Smithsonian Environmental Research Center, Smithsonian Institution, Edgewater, Maryland, United States of America; 3 Cell Biology and Molecular Genetics, Maryland Agricultural Experiment Station, University of Maryland, College Park, Maryland, United States of America; Montreal Botanical Garden, Canada

## Abstract

The tremendous diversity of land plants all descended from a single charophyte green alga that colonized the land somewhere between 430 and 470 million years ago. Six orders of charophyte green algae, in addition to embryophytes, comprise the Streptophyta s.l. Previous studies have focused on reconstructing the phylogeny of organisms tied to this key colonization event, but wildly conflicting results have sparked a contentious debate over which lineage gave rise to land plants. The dominant view has been that ‘stoneworts,’ or Charales, are the sister lineage, but an alternative hypothesis supports the Zygnematales (often referred to as “pond scum”) as the sister lineage. In this paper, we provide a well-supported, 160-nuclear-gene phylogenomic analysis supporting the Zygnematales as the closest living relative to land plants. Our study makes two key contributions to the field: 1) the use of an unbiased method to collect a large set of orthologs from deeply diverging species and 2) the use of these data in determining the sister lineage to land plants. We anticipate this updated phylogeny not only will hugely impact lesson plans in introductory biology courses, but also will provide a solid phylogenetic tree for future green-lineage research, whether it be related to plants or green algae.

## Introduction

It is hard to imagine what the planet looked like 500 million years ago, before green algae first colonized the terrestrial habitat. Plants now blanket the highest alpine peaks, the lowest deserts, tropical rainforests, arctic expanses and even aquatic and marine environments. Microfossils and fragments of plant tissue from the middle Ordovician (458–470 mya) reveal evidence of the first plant colonizers [1], [2], but these pioneering species and their green-algal progenitors have long since disappeared. Descendants of these early pioneers are widespread, however, which begs the question: Which extant green algal group is the closest living relative of land plants?

Despite a decade of molecular phylogenetic research on land plants and green algae, this question is far from settled. Land plants (LP), or embryophytes, are a monophyletic group nested within charophytes, a group of fresh water green algae. Together, the charophytes and embryophytes constitute the monophyletic Streptophyta. The other green algal lineage, the Chlorophyta, contains a diverse assemblage of marine and fresh water green algae. It was nearly a decade ago that Karol *et al.* [3] concluded after a four-gene, three genome analysis that, of the charophytes, the Charales constitute the closest living relative to land plants. Another combined data analysis [4] supported the same topology and, for a time, this appeared to be a settled matter. Over the past century, the Charales-as-sister relationship has been used widely in biology textbooks [5]–[7] and, from a morphological standpoint, this relationship tells a good story: as the charophyte lineages diverge, their body plans grow increasingly complex from unicellular (Mesostigmatales) to sarcinoid packets (Chlorokybales) to un-branched filaments (Klebsormidiales) to branched filaments (Zygnematales), to parenchematous tissue (Coleochaetales) and finally to the macrophytes (Charales). From there, the body plans evolve into early land colonizers equipped with complex tissues allowing life out of water. Similarly, sexual reproduction evolves from isogamy in the ancestral lineages to oogamy into the more derived charophyte lineages.

But in spite of morphological support for Charales as sister to land plants, other data conflict with this interpretation. Plastid gene phylogenies provide support for Zygnematales as sister to land plants [8], [9]. In addition, new data based on nuclear genes [10] support this alternative topology. Zygnematales are conjugating (sexual) green algae with both filamentous and unicellular (but no flagellate) forms.

One explanation for the incongruence between topologies could be taxon sampling; the four-gene topology (Charales+LP) [3] has much broader taxon sampling (26 algal taxa) than the reconstructions supporting Zygnematales+LP (six charophytes each) [9], [10]. There is one study with broader taxon sampling (15 algal taxa [8]) that puts Zygnematales as sister to land plants, but there is much less support for this relationship.

A second alternative topology also has emerged: *Coleochaete*+LP. Molecular data supporting this relationship were derived exclusively from nuclear ribosomal protein genes [11]. While additional characters such as plasmodesmata and a nad5 intron support this topology, Coleochaetales as an order is not reconstructed as monophyletic in this phylogeny, which causes concern for the overall topology.

To address this uncertainty in the field, we sought a comprehensive genome scale analysis using a deep sampling of many genes drawn from seven species distributed across all major charophyte lineages: Charales, Coleochaetales, Zygnematales, Klebsormidiales, and Chlorokybales. In addition, we included published Sanger sequences from a *Mesostigma viride* EST library [12] and analyzed them alongside our in-house transcriptomes. From these data we identified a set of orthologs common across the green lineage (Chlorophyta+Streptophyta) using an unbiased approach (no *a priori* gene selection). This yielded a large set of nuclear encoded protein genes that we used to reconstruct the phylogeny and identify the sister lineage to land plants.

## Results

Our taxon sampling included a total of 14 taxa: eight charophytes, four land plants and two chlorophytes. Five of the charophytes were newly collected transcriptomes (Table 1). Both Sanger sequencing (4,992–5,760 reads per taxon) and 454 GS FLX Titanium sequences (444,743–1,077,311 reads per taxon) were gathered. The assembled raw reads into contigs represent mRNA in the organism at the time of collection. The contigs with a putative coding region, as predicted by ESTscan, were referred to as unigenes. These numbers ranged from 12,697 to 33,106 unigenes per taxon.

**Table 1:** Primary sequence data and summary of clustering results.

	454 reads	5′ Sanger reads	454 clustering	454+Sanger clustering	Unigenes
*Chaetosphaeridium globosum*					
Number of reads	884,238	5,760	58,188	25,165	23,490
Average length (bp)	562	949	513	656	515
C*hlorokybus atmophyticus*					
Number of reads	444,743	4,992	19,801	12,731	12,607
Average length (bp)	513	950	726	903	904
*Klebsormidium flaccidum*					
Number of reads	994,649	4,992	51,855	25,554	24,881
Average length (bp)	538	946	629	849	731
*Penium margaritaceum*					
Number of reads	1,077,311	4,992	76,769	30,499	29,880
Average length (bp)	527	943	571	811	638
*Nitella hyalina*					
Number of reads	949,065	4,992	86,432	42,331	33,106
Average length (bp)	547	955	544	682	492

Unigenes are contigs with a putative coding region.

**Figure 1:**
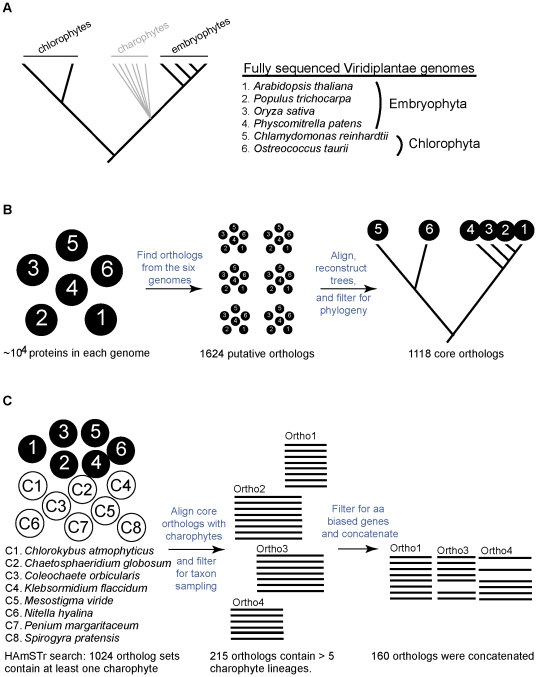
Ortholog identification method. This diagram outlines the steps used for identifying orthologous genes for phylogenetic analysis. **A**) Unresolved phylogenetic scheme relating chlorophytes, charophytes, and embryophytes with a list of the six taxa with fully sequenced genomes used for the core ortholog determination. **B**) Core ortholog prediction from the previous six taxa. **C**) Charophyte orthog prediction. The core orthologs were then used to search for proteins in each of the eight charophyte transcriptomes. We filtered for good taxon sampling and removed orthologs with significant amino acid bias, resulting in 160 aligned proteins. These were concatenated onto one large multigene data matrix for phylogenetic analysis.

The Inparanoid-TC approach to finding core orthologs yielded 1624 putative orthologous groups, that, when filtered for phylogeny, were reduced to 1118 core othologs (Fig. 1.B). HaMStR identified hits in the charophytes for 1024 of the core orthologs and, after filtering for good charophyte taxon representation and removing 55 genes with amino acid composition bias, there were 160 orthologous genes remaining (Fig. 1.C, gene annotation and associated data in Table S1).

**Figure 2:**
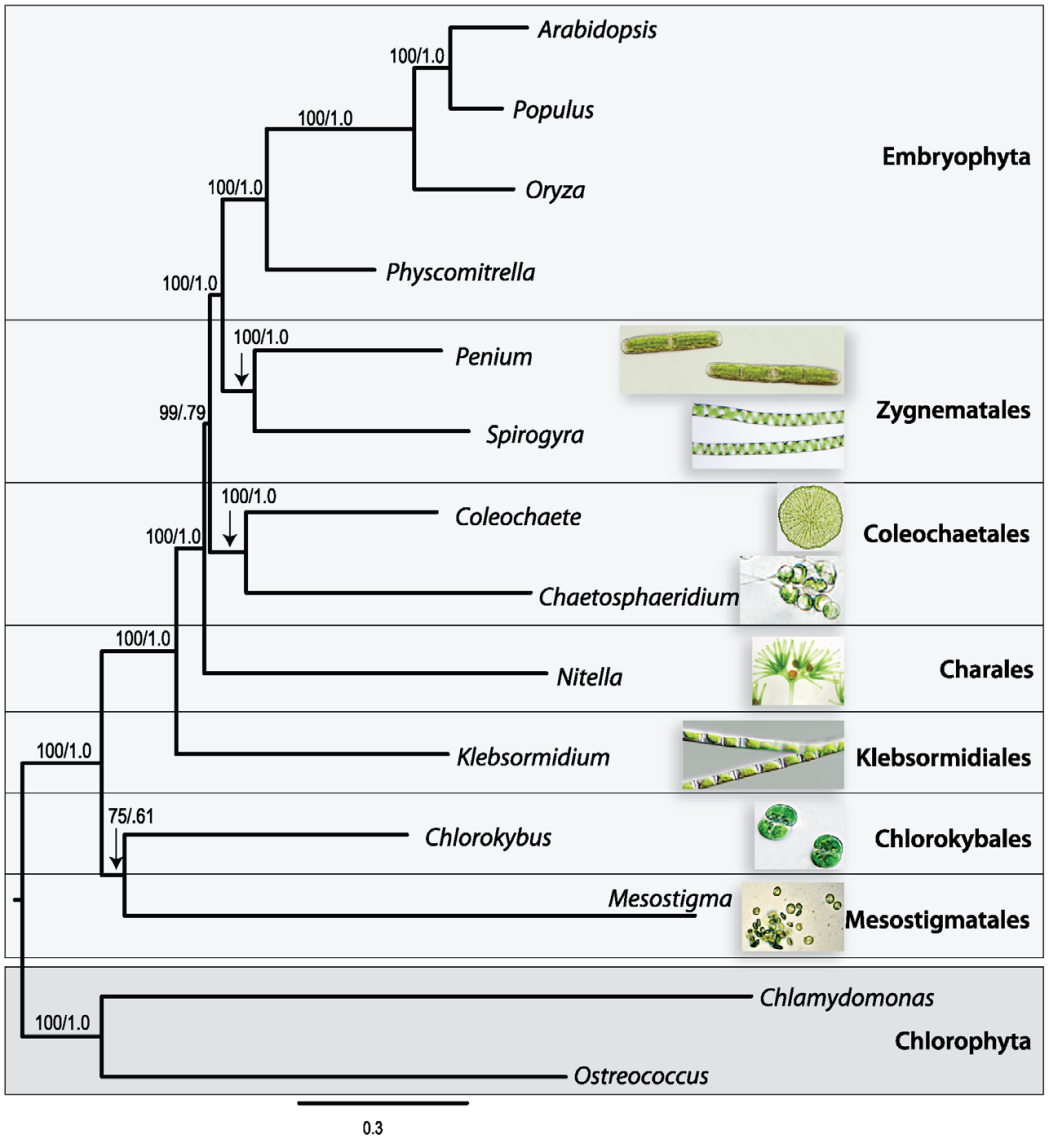
Phylogenetic relationships of 14 Viridiplantae taxa determined by 160 concatenated proteins. Phylogenetic analyses are summarized by a BI (CAT-Poisson model) consensus tree with branch support values from both BI and ML analyses (ML bootstrap/Bayesian posterior probabilities).

We used all 160 orthologous genes to reconstruct the evolutionary history of the 12 streptophytes and two outgroup chlorophytes. To do this, we first concatenated the protein products for 160 genes totaling 99,628 amino acids (46% missing or gapped characters). After trimming for poorly aligned regions, the dataset was condensed to 56,274 amino acids (26% missing or gapped characters). On average, each gene was present in 12 of the 14 taxa, or six of the eight charophytes (Table 2). The representation of individual genes varied among taxa from 65 to 100%, with the exception of *Mesostigma*, which only contained 11% of the 160 genes. This was presumably because of the markedly smaller size of that dataset. Two different phylogenetic analyses were performed on the trimmed alignment; both resulted in the same strongly supported topology (Fig. 2).

**Table 2:** Summary of missing data.

Charophyte taxon	Number of genes	Percent genes
*Chlorokybus atmophyticus*	160	100
*Chaetosphaeridium globosum*	105	65.625
*Coleochaete sp.*	142	88.75
*Klebsormidium flaccidum*	160	100
*Mesostigma viride*	18	11.25
*Nitella hyalina*	160	100
*Penium margaritaceum*	142	88.75
*Spirogyra pratensis*	109	68.125

Genes present for each charophyte taxon in the multigene alignment (160 total).

The ML and BI analyses on the concatenated 160-gene dataset recovered the relationship of Zygnematales as sister to land plants with strong statistical support (ML = 100%, PP = 1.0). The Coleochaetales are sister to the Zygnematales+LP clade (ML = 99%, PP = 0.79) with Charales diverging earlier (ML = 100%, PP = 1.0; followed by *Klebsormidium*: ML = 100%, PP = 1.0). Finally, Chlorokybales and Mesostigmatales are moderately supported as sister to one another (ML = 75%, PP = 0.61), and together they comprise the earliest diverging lineage in the streptophytes (ML = 100%, PP = 1.0). In addition to the branching order of the charophyte lineages, we included two taxa per order for Zygnematales and Coleochaetales. Each was recovered as monophyletic, lending further support for these classically recognized orders.

In large concatenated studies of this type, a logical concern is that a subset of the genes might support alternative topologies. For the most part, this is ignored in multi-gene phylogenetic analyses. But given the propensity of plant phylogenies to have gene-tree/species-tree conflicts [13], we addressed this issue directly by statistically testing our data for incongruence using the program Concaterpillar [14]. Given a multi-gene dataset, this analysis uses a likelihood-ratio test to identify compatible partitions. The program groups genes into sets that are ‘incongruent,’ which Leigh et al. define as genes as having “phylogenetic incompatibility, either due to truly different evolutionary history, or to systematic error” [14]. Fifteen sets ranging in size from 37 to 3 genes (Fig. S1) were identified from our total set of 160 genes. None of these partitions placed Charales as sister to land plants. Not surprisingly, the largest set of 37 genes supported the Zygnematales+LP relationship, which also occurred across four additional sets totaling 71 genes (S1.d, S1.h, S1.k, S1.l) (these sets differed in their placement of *Mesostigma* and other basal charophytes). One noteworthy minority partition recovered the Coleochaetales+LP topology (Fig. S1.c), and two others had *Coleochaete*+LP, with *Chaetospheridium* branching earlier (Fig. S1.j, S1.o).

To ensure we were not tossing phylogenetically informative characters when we eliminated the 55 genes with an amino-acid composition bias, we performed similar phylogenetic analyses on the 215-concatenated-gene set. The resulting ML topology was almost exactly the same, with 100% bootstrap support on every bipartition except for the *Chlorokybus*+*Mesostigma* lineage, where 73% support was recovered. However, the Concaterpiller analyses on this larger gene set recovered an interesting gene set: one of the 15 recovered sets contained 24 genes that supported the *Nitella*+LP topology. The 55 genes with amino acid composition bias were fairly well distributed across the various incongruent sets, but eight of them landed in the Nitella+LP set. This set/topology was not recovered in the subsequent 160-gene Concaterpiller analysis.

## Discussion

This study, which includes all charophyte lineages provides a robust, well-supported result that LP and Zygnematales are sister lineages. We believe our results warrant serious reconsideration of charophyte evolution given that the phylogenomic approach of our study confirms the plastid-encoded analyses of Turmel *et al.* [9] and the recent nuclear-genomic study of Wodniok et al. [10]. Some studies using a targeted gene approach [3], [11], [15], [16] reconstruct alternate topologies, but none has the broad and unbiased nuclear genome sampling used in the current study.

Two phylogenetic studies [10], [11] published in the past year use next-generation sequence data to address a similar question as posed in this manuscript. However, the data collected and analyzed for these studies are almost completely non-overlapping, and consequently the three independent analyses provide diverse perspectives on a difficult and deep evolutionary relationship. Finet et al. [11] focused on 77 ribosomal genes (12,149 characters) that were selected *a priori* from the same transcriptomes collected in this study. Despite the fact that both the present study and that of Finet et al. drew from the same transcriptomic dataset, only five genes overlap in the two studies (out of 1118 core orthologs and 160 selected for the final dataset). Thus, the analyses are almost completely independent. Their tree topology differs from ours with the assignment of *Coleochaete* as the sister lineage to land plants. In addition, it is noteworthy that like the ribosomal-protein tree, ribosomal RNA gene trees do not reconstruct a monophyletic Coleochaetales [17], which – if the Coleochaetales are in fact monophyletic as indicated by morphology and organellar data – suggests that some form of molecular coevolution may underlie this apparent conflict. The other noteworthy study of charophyte phylogenetics came from Wodniok et al. [10]. This is also a broad transcriptomic analysis, but like the Finet et al. study, it makes use of an *a priori* set of selected genes, and draws from a smaller number of charophyte taxa (six), and fewer aligned characters (30,270 amino acids) than our study. While not directly comparable, the Wodniok et al. [10] tree topology is congruent with ours, but with lower branch support on most of the charophyte nodes. The analysis reported here was based on a filtration of roughly 5×10^9^ characters – selecting only for evidence of orthology and combinability – which resulted in a dataset of 99,628 characters, and a strongly supported tree topology. What ultimately sets our analysis apart, however, is that we did no *a priori* gene selection. Thus, in addition to the intrinsic phylogenetic interest, we demonstrate a powerful new approach to data selection that leverages the use of high-throughput sequence data.

However, given the genomic-scale of the data collection, our taxon sampling is limited and may be a source of error [11]. Without additional transcriptomes, we cannot directly test this issue. But long branch attraction is much less a factor when amino acid data are used with an appropriate model of evolution [18], [19]. While short internal branches have been shown to be a source of phylogenetic inconsistency [18], this is a much harder issue to address. Two analyses suggest taxon sampling might not be a confounding issue in this study: 1) the Turmel *et al.* [8] rRNA plastid phylogeny with twice our taxon sampling recovered the Zygnematales+LP relationship using a nucleotide based analysis, and 2) the Charales+LP relationship still emerged when a reduced Karol *et al.* [3] dataset was reanalyzed to approximate our taxon sampling. This second line of evidence provides tenuous support at best but is worth reporting due to its similar taxon spread.

**Figure 3:**
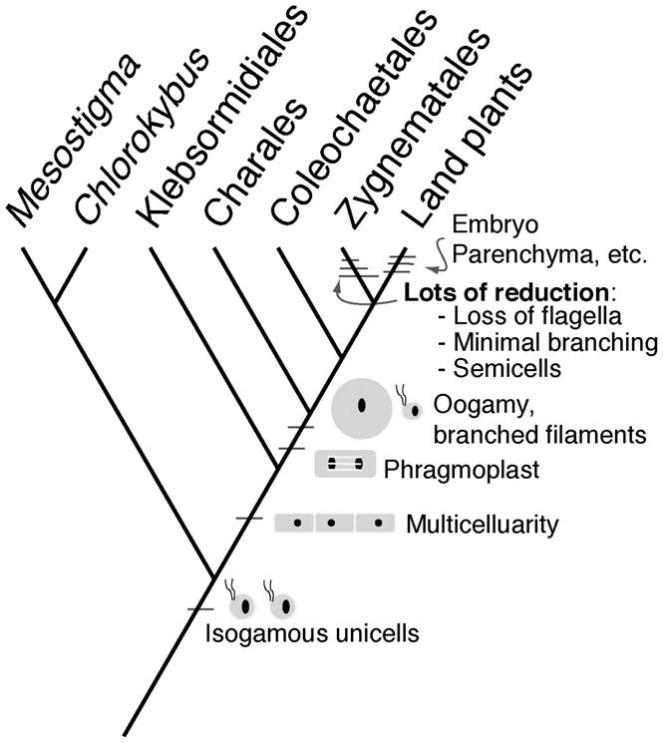
Hypothesis of character evolution in the Charophytes. The earliest branching streptophytes (*Mesostigma* and *Chlorokybus*) were unicellular, flagellate, and isogamous. Multicellularity in the form of unbranched filaments evolved in the common ancestor of the remaining streptophytes and is represented in the Klebsormidiales. The most recent common ancestor of Charales+Coleochaetales+Zygnematales+LP most likely was an alga with plant-like cell division (phragmoplast), branched filaments, and oogamous sexual reproduction. The Charales went on to independently evolve a complex macrophytic form. The Coleochaetales independently acquired parenchymatous tissue and maternally retained zygotes. However, the Zygnematales went the route of reduction: loss of flagellate cells (reproduction via conjugation), loss of multicelluarity (Desmids), and loss of the phragmoplast.

The well supported land plant + Zygnematales topology uses a large suite of genes and requires a rethinking of character evolution in charophyte lineages leading up to land colonization. Previous hypotheses of increasing morphological complexity [20], [21] are not congruent with the results of our study. However, multiple gains and losses of multicellularity across all green algae have been well documented, as has the reduction of characters in the Zygnematales [22], [23]. The Zygnematales include filamentous and unicellular organisms, but the unicellular state may well be a derived condition [23] from branched filamentous ancestors, just as flagellate stages were lost in this order. In this context, it is not a stretch to imagine character reduction in the sister lineage to land plants (Fig. 3) resulting in the loss of homologous characters potentially shared in the common ancestor. The multicellular complexity in Charales and Coleochaetales appears to be independently derived from a common branched and filamentous ancestor, one likely to have had oogamous reproduction. These characters were probably present in the common ancestor of all four “advanced” lineages, an idea that has been suggested by previous investigators [24]. In this model, however, the parenchyma-like organization, axial growth and protonema of Charales would be examples of parallel evolution, as would the multiple zygotic products of *Coleochaete*.

In conclusion, our research lends strong support to the notion that the closest living green algal lineage to land plants is not the plant-like stoneworts (Charales) as previously thought, but a species-rich assemblage of fresh-water filamentous and unicellular organisms, better known as pond scum.

## Materials and Methods

### Algal sampling

All seven transcriptomes were similarly processed (see Timme and Delwiche [25] for detailed methods on *Spirogyra pratensis* UTEX 928 and *Coleochaete sp.* CFD). In summary, *Chaetosphaeridium globosum* SAG 26.98, *Penium margaritaceum* SKD2004_CL18 (culture available from David Domozych, Skidmore College, Saratoga Springs, NY), *Klebsormidium flaccidum* UTEX 321 and *Chlorokybus atmophyticus* UTEX 2591were grown up in appropriate culture media 18°C and a 12∶12 LD photoperiod with a photon flux of 180–200 µmol s-1 m-2. *Nitella hyalina* KGK0190 (culture available from Kenneth Karol, The New York Botanical Garden, Bronx, NY) was cultured in a fresh water aquarium at room temperature. Cultures were harvested during log phase growth in a variety of conditions to maximize the diversity of transcripts: at intervals of 7 am, 12 pm, 4 pm and 9 pm; after sitting in a dark enclosure for 24 hours; and after being exposed to 20 minutes of −20°C. Algal cultures were pelleted at 4000rpm (*Nitella* did not require centrifugation), dropped in liquid nitrogen and stored at −80°C until RNA extraction.

### RNA isolation

Frozen tissue was ground at cryogenic temperatures using a SPEX 6770 Freezer/Mill (SPEX Certi Prep, Metuchen, NJ). The ground cells were then added to Tri Reagent (Molecular Research Center, Inc., Cincinnati, OH), where the manufacture's protocol was followed. Extra chloroform extractions and an additional LiCl precipitation were required to eliminate polysaccharide and genomic DNA contamination. After each isolation, the nucleic acid concentration and OD ratios were quantified with a NanoDrop (Thermo Scientific NanoDropTM 1000 Spectrophotometer, Wilmington, DE) and the quality of RNA, was determined by running 1 µg of total RNA on a 1.2% agarose MOPS/formaldehyde gel (Applied Biosystems/Ambion, Austin, TX) stained with ethidium bromide, then examining the rRNA banding patterns. High-quality, clean RNA was pooled until 1 mg of total RNA was reached.

### cDNA construction and DNA sequencing

Total RNA (∼1 mg) was shipped on dry ice to Agencourt Bioscience Corporation (Beverly, MA) where Poly(A)+RNA from total RNAs was isolated, converted to double stranded cDNA, size fractionated (<1.2 kb), cloned directionally into the pExpress 1 vector and grown up in T1 phage resistant *E. coli*. Subsequent DNA sequencing included both 5 prime Sanger reads and 454 sequencing technologies. In summary, each taxon had 5,000–10,000 Sanger reads plus a full plate of GS FLX Titanium 454 sequences generated (see Table 1 for exact numbers). For the Sanger sequencing, DNA from the clones was purified using Agencourt's proprietary solid-phase reversible immobilization (SPRI) system. The purified DNA was then sequenced using ABI dye-terminator chemistry and run on ABI 3730 (Applied Biosystems Inc, Foster City, CA) machines. In addition, we included published Sanger sequences for one additional taxon, *Mesostigma viride* [12]. For the 454-sequencing, 3–5 ug of isolated DNA was nebulized to a mean size range of 3–500 bp, followed by a size selection of fragments >300 bp by column exclusion and Ampure™ (Agencourt Bioscience, Danvers, MA) isolation. Adapters were ligated onto the fragments and selected using library capture beads. The single stranded fragments were isolated followed by standard library dilutions. The library was amplified onto DNA capture beads by emulsion PCR (emPCR). DNA capture beads were collected and a sequencing primer was annealed by a thermocycler. Beads for each genome were placed on the picotitre plate, sequenced on the Roche 454 GS FLX instrument, and analyzed with base-calling software using default parameters.

### Transcriptome clustering method

The clustering for each taxon was performed in a two-step process. First, the 454 reads were clustered using MIRA vs 2.9.43 [26]. Second, the raw Sanger reads were combined with the 454 contigs and respective quality scores and processed through the EST2uni pipeline [27], which used a variety of methods to remove low-quality sequence, vector contamination and low complexity regions. It then clustered the clean reads with CAP3 [28] using a 100 bp plus 95 percent identity of overlap. ESTscan [29] predicted the protein-coding regions in the contigs and singletons using *Arabidopsis thaliana* score matrix. The clustering process resulted in a set of predicted proteins, or unigenes, for each taxon, which were then used for all downstream analyses.

### Ortholog prediction using extended HaMStR approach (Fig. 1)

The HaMStR approach [30] to ortholog prediction uses a well-curated set of genes, or ‘core orthologs’, to identify putative orthologs from an EST library. For each core ortholog, HaMStR searches a set of unigenes and identifies a set of putative orthologs, if present. Because no curated set of orthologs exist for the entire green lineage, we set about building our own. Six fully sequenced genomes were chosen to construct the core orthologs: four embryophytes and two chlorophytes (Fig. 1.A): *Arabidopsis thaliana* (Uniprot v. 1.0), *Populus trichocarpa* (JGI v. 1.1), *Oryza sativa* (Plantbiology v. 1.0), *Physcomitrella patens* (JGI v. 1.1), *Ostreococcus tauri* (JGI v. 2.0) and *Chlamydomonas reinhardtii* (JGI v. 3.0). The phylogenetic positions of the core ortholog taxa were ideal for our purposes – unless there was gene loss, any ortholog present in both embryophytes and chlorophytes also should be present in charophytes. The protein sequences for each of the six genomes were used to infer the set of core orthologs using a modified Inparanoid [31] approach, Inparanoid-TC [30]. Because genome duplication in embryophytes can cause paralogy issues, we used a phylogenetic filter to confirm true orthology (Fig. 1.B). Briefly, we aligned each putative orthologous group using Muscle [32], [33], trimmed each alignment with trimAl (gt = 0.4, w = 3, st = 0.01) [34], reconstructed the Maximum Likelihood (ML) phylogeny using RAxML [35], [36] (f = a, # = 100, m = PROTGAMMAWAG), and used an in-house perl script to run the PAUP [37] ‘filter’ command, identifying the ML topologies consistent with well-known phylogenetic relationships. The orthologs that passed this filter were considered our core orthologs (Fig. 1.B).

These Viridiplantae core orthologs then were used as input to the program HaMStR. Instead of identifying a set of orthologs in each transcriptome, we modified the HaMStR program to extract the top hit only so that, if present, we had a single putative ortholog for each of the eight transcriptomes. This modification allowed us to submit the top hit directly into a phylogenetic analysis. After all eight HaMStR analyses were preformed and alignments were made using Muscle, we gathered the set of core orthologs that had at least one match in the charophytes (Fig. 1.C). Because these orthologs were collected for phylogenetic purposes, we filtered for good taxon sampling: at least one charophyte for each major charophyte lineage, or *Chlorokybus*, *Klebsormidium*, *Nitella*, *Coleochaete* OR *Chaetosphaeridium*, and *Penium* OR *Spirogyra* (Fig. 1.C).

And lastly, because these genes span such divergent taxa (up to one billion years divergence time), changes in amino acid compositional heterogeneity over time was an issue we wanted to minimize. In this spirit, we used TREE-PUZZLE [38] to identify orthologs with significant amino acid bias. An assumption of any phylogenetic analyses assumes that the character composition does not change over time; so removing genes that have a significant amino acid bias eliminated a possible source of systematic error. This last filtering step produced a set of aligned orthologous genes that had good taxon sampling and no amino acid composition bias (Fig. 1.C). These were concatenated onto one large multi-gene data matrix (detailed in the following section).

### Reconstructing the multi-gene phylogeny

We aligned the amino acids for each unigene using Muscle [32], [33] (default parameters), concatenated them using an in-house perl script, trimmed poorly aligned regions using trimAl (gt = 0.4, w = 3, st = 0.01) [34], estimated the model of evolution for the ML analysis using ProTest2.4 [39], and ran phylogenetic analyses on the multi-gene dataset: Maximum Likelihood (ML) (LG+G+F model) using RaxML [36], [40] and Bayesian Inference (BI) (CAT-Poisson model) using PhyloBayes [18], [41], [42]. The BI analysis allowed us to test the effect of applying a site-heterogeneous model of evolution (CAT) [18] to our multi-gene amino acid data matrix. To measure phylogenetic stability, bootstrapping was performed for the ML analysis and posterior probabilities (PP) were inferred by BI analysis.

### Data access

The individual reads for each transcriptome were deposited in GenBank, http://www.ncbi.nlm.nih.gov/. The Sanger reads are located in dbEST under the following accession numbers: *Chlorokybus atmophyticus* (GenBank: HO407395-HO431109), *Chaetosphaeridium globosum* (GenBank: HO348296-HO407394), *Klebsormidium flaccidum* (GenBank: HO431110-HO486407), *Nitella hyalina* (GenBank: HO486408-HO574687), and *Penium margaritaceum* (GenBank: HO574688-HO651665). The 454 sequences are in the Sequence Read Archive (SRA): *C. atmophyticus* (GenBank: SRX025846.1), *C. globosum* (GenBank: SRX025844.1), *K. flaccidum* (GenBank: SRX025847.1), *N. hyalina* (GenBank: SRX025843.1), and *P. margaritaceum* (GenBank: SRX025845.1). The clustered 454+Sanger reads are deposited in the Transcriptome Shotgun Assembly Sequence Database (TSA): *C. atmophyticus* (GenBank: JO192127 - JO204622), *C. globosum* (GenBank: JO157958 - JO182157), *Coleochaete sp.* (GenBank: JO233843 - JO252228), *K. flaccidum* (GenBank: JO252229 - JO277141), *N. hyalina* (GenBank: JO277142 - JO317756), *Spirogyra pratensis* (GenBank: JO182540 - JO192126) and *P. margaritaceum* (GenBank: JO204623 - JO233842). The trimmed alignment, ML tree and BI consensus tree were uploaded to TreeBase and are accessible from the following URL: http://purl.org/phylo/treebase/phylows/study/TB2:S10897.

## Supporting Information

Figure S1 **Concaterpillar ML trees derived from compatible partitions of the multigene alignment.** Set numbers were determined by Concaterpillar and are listed in the figure by descending size.
(TIF)

Table S1 **Tab-delimited text file containing annotation and summary data for the 160 orthologs used in the phylogenetic analysis.**
(TXT)
