# Supplementary figures and images for: Broad Phylogenomic Sampling and the Sister Lineage of Land Plants
Source: PLoS One. 2012 Jan 13;7(1):e29696. doi: 10.1371/journal.pone.0029696 (PMC3258253; doi:10.1371/journal.pone.0029696)

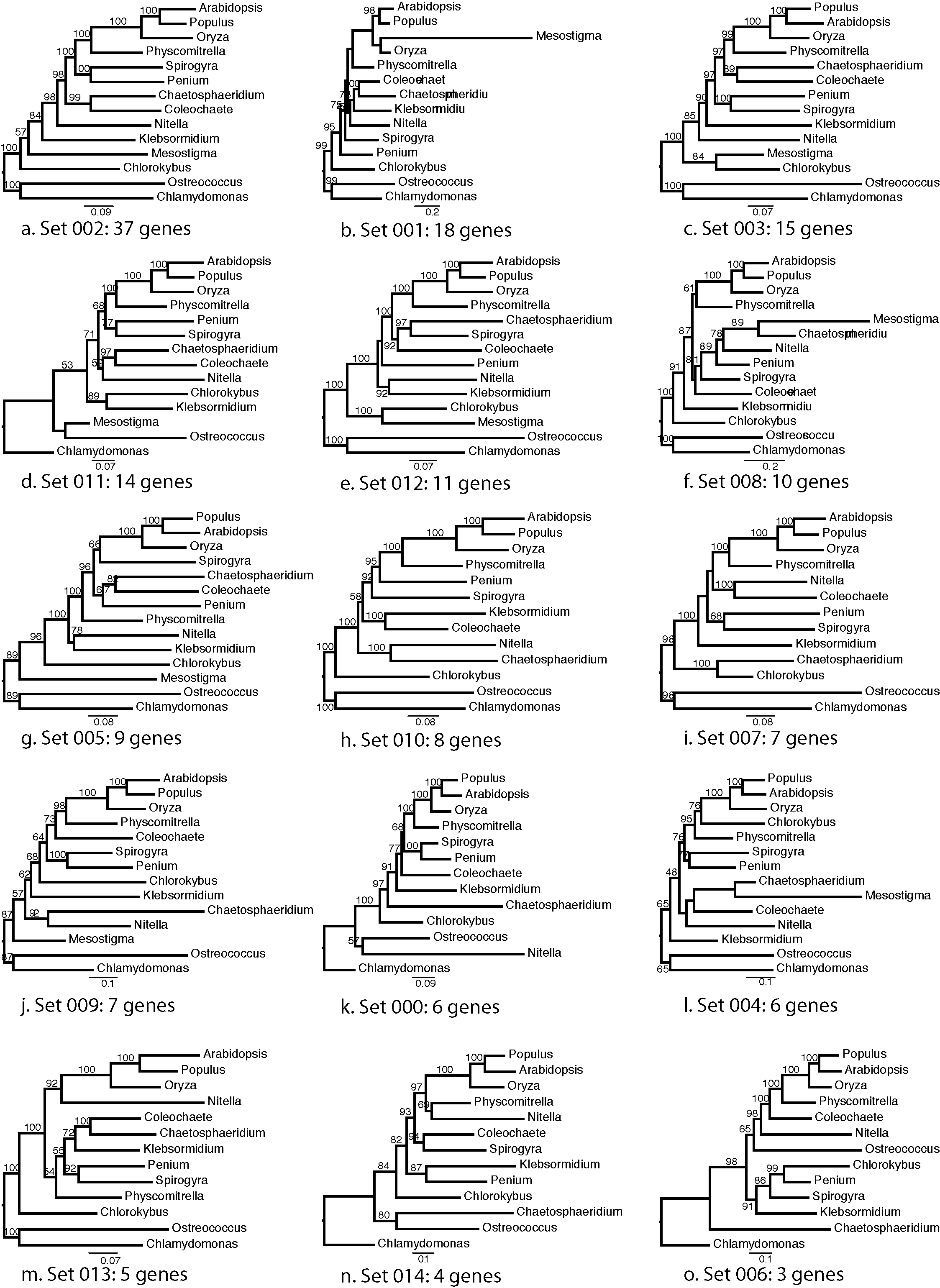

Supplement: Figure S1 — Concaterpillar ML trees derived from compatible partitions of the multigene alignment. Set numbers were determined by Concaterpillar and are listed in the figure by descending size. (TIF) [file pone.0029696.s001.tif]
